# The beneficial effects of simultaneous supplementation of *Lactobacillus reuteri* and calcium fluoride nanoparticles on ovariectomy-induced osteoporosis

**DOI:** 10.1186/s12906-023-04167-6

**Published:** 2023-09-26

**Authors:** Dibachehr Rahmani, Bahareh Faal, Hakimeh Zali, Saeed Hesami Tackallou, Zahra Niknam

**Affiliations:** 1 Department of Biology, Central Tehran Branch, Islamic Azad University, Tehran, Iran; 2 Department of Tissue Engineering and Applied Cell Sciences, School of Advanced Technologies in Medicine, Shahid Beheshti University of Medical Sciences, Tehran, Iran; 3 Neurophysiology Research Center, Cellular and Molecular Medicine research Institute, Urmia University of Medical Sciences, Urmia, Iran

**Keywords:** Bone biomarkers, Calcium fluoride nanoparticles, *Lactobacillus reuteri*, Osteoporosis, Ovariectomy

## Abstract

**Background:**

The development of new strategies to inhibit and/or treat osteoporosis as a chronic systemic disease is one of the most crucial topics. The present study aimed to investigate the simultaneous effects of calcium fluoride nanoparticles (CaF_2_ NPs) and *lactobacillus reuteri* ATCC PTA 6475 *(L. reuteri*) against osteoporosis in an ovariectomized rat model (OVX).

**Methods:**

In this study, 18 matured Wistar female rats were randomly assigned into 6 groups, including control, OVX, sham, OVX + *L. reuteri*, OVX *+* CaF_2_ NPs, and OVX + *L. reuteri* + CaF_2_ NPs. We used OVX rats to simulate post-menopausal osteoporosis, and the treatments were begun two weeks before OVX and continued for four weeks. All groups’ blood samples were collected, and serum biomarkers (estrogen, calcium, vitamin D_3_, and alkaline phosphatase (ALP)) were measured. The tibia and Femur lengths of all groups were measured. Histopathological slides of tibia, kidney, and liver tissues were analyzed using the Hematoxylin and Eosin staining method.

**Results:**

Our results revealed that dietary supplementation of *L. reuteri* and CaF_2_ NPs in low doses for 6 weeks did not show adverse effects in kidney and liver tissues. The tibial and femoral lengths of OVX rats as well as the population of osteoblasts and osteocytes and newly generated osteoid in the tibia remarkably increased in the combination therapy group. Moreover, there was a significant increase in serum estrogen levels and a significant decrease in serum calcium and alkaline phosphatase levels in combination treatment groups compared to the OVX groups not receiving the diet.

**Conclusions:**

Our results suggest the favorable effects of the simultaneous supplementation of *L. reuteri* and CaF_2_ NP to reduce post-menopausal bone loss.

**Graphical abstract:**

**Figure:**
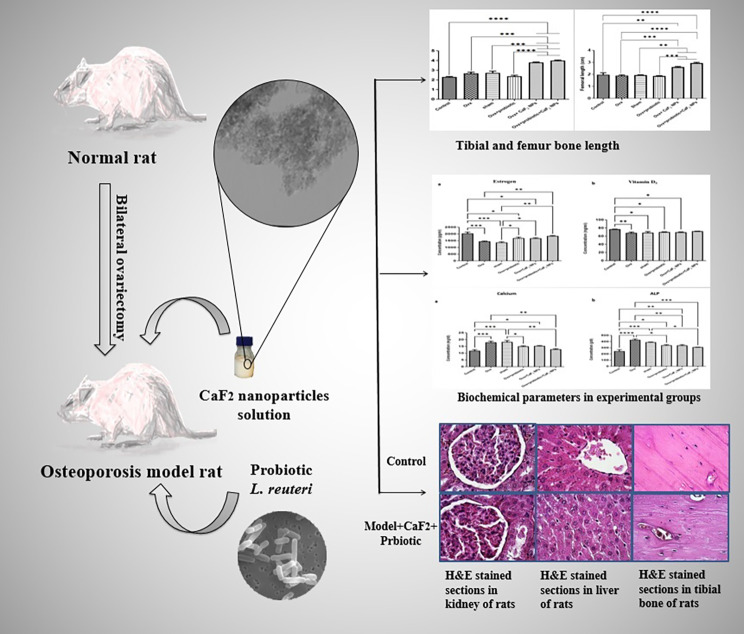

## Introduction

Osteoporosis is one of the major life-threatening diseases that impact over 200 million people worldwide and mostly affects women [1]. This skeletal disorder is identified by diminished bone strength, low bone mass, and deterioration of bone tissue, leading to an increased risk of fractures [2]. Aging and the associated increase in reactive oxygen species (ROS) cause an imbalance between reabsorption and bone formation rate, resulting in an increased risk of osteoporosis [3]. Postmenopausal women experience a rapid phase of bone mass reduction due to ovarian malfunction and estrogen deficiency, as a result, the bone fracture rate in women is 2 to 3 fold higher than in men [2]. Loss of estrogens or androgens reduces defense against oxidative stress in bone, resulting in enhanced bone resorption [4].

For the prevention of osteoporosis, it has been recommended for elderlies to take daily doses of 1200–1500 mg of calcium and 400–800 IU of vitamin D_3_ as they should be taken concurrently. Nowadays, several therapeutic strategies for osteoporosis are presented, such as anabolic and catabolic treatments and treatments using biomolecules and biomaterials [5–8]. However, most of the medications used for osteoporosis treatment show several adverse effects. For example, bisphosphonates are the most effective class of drugs in the treatment and prevention of osteoporosis. However, this class of drugs also has side effects, including gastrointestinal complications such as gastritis or esophageal inflammation, abdominal pain, nausea, diarrhea, and constipation [9]. In addition, bisphosphonates can inhibit osteoclast activity resulting in abnormal bone remodeling that can lead to irregular femur fractures or osteonecrosis of the jaw in some patients [1]. Another more effective treatment for inhibiting bone loss is hormone replacement therapy, such as Estrogen replacement therapy (ET) and estrogen plus progesterone therapy (EPT). Estrogen-based drugs are divided into natural medicines, such as estradiol and synthetic drugs, such as ethinylestradiol. While estrogen replacement therapy can decrease the risk of bone loss and osteoporotic bone fractures during menopause, long-term use of this therapy can enhance the risk of serious illnesses such as venous thromboembolism and invasive breast cancer [1, 10]. Receptor activator of nuclear factor kappa-Β ligand (RANKL) inhibitors, such as Denosumab, reduce the risk of vertebral and hip fractures and enhance bone mineral density (BMD) across skeletal sites [11]. Despite the potential effect of Denosumab on postmenopausal women with an increased/high risk of osteoporosis, discontinuation of treatment may lead to a rapid reduction in BMD and multiple vertebral fractures [10]. Treatments based on stem cells can enhance cell lifespan and activity to reverse degenerative damage to the regeneration of bone. Despite its potential to promote bone formation, stem cell therapy has shortcomings, such as ethical issues in human research and their potential for unwanted differentiation and to suppress antitumor immune responses [12]. miRNA-based therapy as another treatment for osteoporosis showed significant potential [13]. For instance, in a study on ovariectomized (OVX) rats, the BMD of rats treated with miR-30a-3p inhibitors was significantly increased compared with the OVX group [14]. However, miRNAs might affect multiple signaling pathways that could lead to adverse effects of off-target activities [10]. Despite the existence of various drugs, the existing treatments for osteoporosis have little effect and even in some cases have a doubtful effect, which may even have a negative role in the long run. Today, as the world’s population ages, the social and economic problems of osteoporosis are on the rise. Therefore, finding a suitable treatment with fewer side effects is a priority.

Gut microbiota plays a key role in calcium and vitamin D metabolism, and also it has been proved that the gut microbiome regulates bone homeostasis in mice, potentially through modulating the immune system and osteoclast generation, proposing that modulating the microbiome and/or inflammatory tone may contribute to regulate bone mass and inhibit osteoporosis [15]. Recently, great attention has been given to the administration of probiotics which are defined as health-promoting live microorganisms and have beneficial impacts on host health and disease inhibition and/or treatment when used in enough amounts [16]. The efficacy of multiple probiotic strains in enhancing bone health has been indicated in several animal models [1]. *lactobacillus reuteri* (*L. reuteri*) is one of the nonpathogenic probiotic bacterium that produces useful immunomodulatory agents. Inflammation causes accelerated bone loss because of the induction of osteoclastogenesis and enhanced bone resorption [15]. In the human body, *L. reuteri* is found in different areas, such as the skin, the urinary tract, the gastrointestinal tract, and breast milk [17]. A previous study demonstrated that *L. reuteri* treatment significantly decreased osteoclastogenesis and bone loss in OVX mice [18]. Another study showed that *L. reuteri* can reduce intestinal inflammation and enhance bone density in healthy male mice [19]. Since bone is a calcium reservoir in the body in different forms, including hydroxyapatite (HA) (85%), calcium carbonate (10%), magnesium fluoride (3%), and calcium fluoride (CaF_2_) (3%) [20], nano-calcium supplements such as CaF_2_ can reduce the risk of osteoporosis. For example, the results of a study showed that injections of nano-calcium carbonate and nano-calcium citrate maintain the bone mineral density of the whole body in OVX rats [21]. Fluoride, the ionic form of fluorine, also has the greatest potential as a therapy for osteoporosis and is identified to promote osteoblast differentiation [22]. The rate of vertebral fractures in patients with postmenopausal osteoporosis was measured after receiving a combination of calcium, fluoride, and estrogen. It was found that this combination therapy is more effective for reducing vertebral fractures [23]. OVX in rats leads to bone loss related to altered immune status, like post-menopausal osteoporosis. Due to the effectiveness of the treatment performed by nanoparticles and compounds containing calcium and fluoride on osteoporosis as well as the positive effects of *L. reuteri* on bone health in research, this experiment was designed to determine if *L. reuteri* ATCC PTA 6475 and CaF_2_ NPs treatments protect rats from OVX-induced bone loss.

## Materials and methods

### Experimental design

Eighteen adult female Wistar rats (200–250 g) 6 weeks of age were obtained from the laboratory of Islamic Azad University Central Tehran Branch and applied according to laboratory animal bioethical principles and guidelines [24]. They had access to standard amounts of food, water, and space. They were also acclimated to the animal room for 1 week before the start of the experiment. The environment was maintained at 22 ± 2°C with a 12-hour light/dark cycle. The rats were fed a standard chow consisting of carbohydrate 48.8%, protein 21%, fat 3%, calcium 0.8%, phosphorus 0.4%, fibre 5%, moisture 13%, and ash 8% [18, 25]. All animal handling and procedures followed the international guidelines for the use and care of laboratory research animals and were according to the guidelines of the Institutional Animal Care and Use Committee of Islamic Azad University Central Tehran Branch. In this study, we used OVX rats to simulate post-menopausal osteoporosis and the experiment and treatments were begun two weeks before OVX and continued for four weeks. Animals were randomly divided into six groups to minimize bias; each group containing 3 rats, including Control (group 1): non-OVX untreated rats; OVX (group 2): ovariectomized untreated rats; sham (group 3): OVX rats treated with 300 µl blank solution (PBS) for experiencing gavage stress; OVX + *L. reuteri* (group 4): OVX rats treated with 300 µl solution, which contains 100 µl probiotic *L. reuteri* ATCC PTA 6475 (Takgene Zist Company, Iran) (1 × 10^9^ colony forming units (CFU)/ml in PBS buffer and 200 µl PBS; OVX + CaF_2_ NPs (group 5): OVX rats treated with 300 µl solution, which contains 200 µl CaF_2_ NPs solution (1 mg/ml) and 100 µl PBS, and OVX + *L. reuteri* + CaF_2_ NPs (group 6): OVX rats treated with 300 µl solution, which contains 100 µl probiotic *L. reuteri* (1 × 10^9^ CFU/ml) and 200 µl of CaF_2_ NPs solution (1 mg/ml). Oral gavage was performed with a stainless steel feeding needle for oral administration of medications in rats. This Animal research is reported in accordance with “ARRIVE” guidelines.

The bacterial solution dosage was determined according to the previous studies [18, 25, 26], which did not observe any side effects in this dose. Also, the daily CaF_2_ NPs dosage was chosen at 1 mg/ml, which was tested on “Vero cells” in another study and had no cytotoxic effects [27].

### Calcium fluoride nanoparticles preparation

To synthesize calcium fluoride nanoparticles (CaF_2_ NPs) via the co-precipitation method, pure CaCl_2_ (Scharlau, molecular mass = 110.984) and NH_4_F (Merck, molecular mass = 37.036) were used [27]. Firstly, we dissolved 0.01 mol CaCl_2_ in 100 ml distilled water, following this, 0.02 mol of NH_4_F was added to the solution under vigorous stirring via a magnetic stirrer for 2 h. The mixed solution gradually transformed into an opaque white suspension during stirring. Then, the prepared suspension was centrifuged at 3000 rpm for 10 min and washed five times with Ethanol by centrifugation to eliminate the ammonium ions and the residual chloride.

### Characterization of CaF_2_ NPs

The typical X-ray diffraction (XRD) spectra of the synthesized CaF_2_ NPs was determined by an X-ray diffractometer (Cu Kα line λ = 0.15406 nm). The intensity was identified in the ranges of 10° < 2θ < 90°, with 0.07° step size [28]. Inductively Coupled Plasma-Optical Emission Spectroscopy (ICP-OES, Model: ICP-OES 730-ES, Varian) was employed to investigate the calcium content in the nanoparticles solution. The concentrations of calcium and fluoride ions in the sample were measured by X-ray fluorescence (XRF) (Philips PW1606) analysis. Dynamic light scattering (DLS) was also used to determine the hydrodynamic size of CaF_2_ nanoparticles using a VASCO PARTICLE SIZE ANALYZER software: NanoQ V2.5.4.0. The nanostructure of the particles was assayed by Transmission Electron Microscopy (TEM, Zeiss EM900) at an accelerating voltage of 120.0 kV. The TEM sample was prepared via coating sample onto a 200 mesh carbon-coated Cu-grid; a well-sonicated dilute suspension of sample was used in acetone to reduce agglomeration.

### Surgical procedure (ovariectomy)

Osteoporosis modeling was performed on rats using a bilateral ovariectomy method. The anesthetized rodents (using xylazine and ketamine in concentrations10 mg/kg and 100 mg/kg, respectively) were laid on the surgical table and fixed by staying plaster. The bulged area on the back of the rat’s body was shaved on both sides. The skin incision site was chosen in the most bulging position on the back. A cut was made to the length of 1.5 cm on the skin to show the dorsolateral abdominal muscles. Entrance to the abdominal cavity was achieved by detaching muscles and tissues, which revealed the adipose tissue around the ovary. The upper tissues, like adipose and muscle, were pulled away until the ovary and uterine tube appeared underneath. The distal uterine horn was identified as the incision site to cut the ovarian tissue. The uterine tube was returned to the abdominal cavity, and the muscle and skin were stitched [29]. After the surgery, a morphine ampoule (5 mg/kg S.C) was injected into the rats, and they were kept in separate racks for a few days until the surgical site thoroughly healed. Also, oxytetracycline spray was used to prevent any infections [25].

### Growth and body weight

Rat’s weights have been measured at the beginning of every week and the results have been compared between all the groups. At the beginning of the trial, rats weighed around 200–250 g.

### Serum biochemistry parameters analysis

Blood samples were taken from the rats at the end of the treatments. About 5 mL blood sample was taken by heart tapping from each rat under anaesthetic, and then they were sacrificed instantly [30]. After clotting at room temperature for 5 min in coagulation tubes, serum samples were centrifuged for 10 min at 4000 rpm at 4 ˚C [18], and the supernatant above blood cells was collected for detection biochemical markers. Blood serum biomarkers analysis was performed according to the manufacturer’s instructions of bioassay kits for measurement of Estrogen (Estrogen (Rat) ELISA Kit K4266 BioVision, Inc), Calcium (CALCIUM ARSENAZO), Vitamin D_3_ (25-OH-Vitamin D KIT, Bti) and Alkaline Phosphatase (ALP, Pars Azmoon Company, Iran).

### Measurement of bone length

6 weeks after the start of the treatment, the rats were sacrificed and the femur and tibia were isolated from the leg and the muscle tissues were cleaned, the sizes of both bones were determined with a digital caliper [31].

### Hematoxylin and eosin (H&E) staining

Rats’ kidneys, liver, and bone were sampled. Processing of tissue samples for histological examinations followed established methods. In brief, tissue samples were rinsed with phosphate-buffered saline (PBS) and fixed in 10% formalin. Fixed tissues were embedded in paraffin, and then deparaffinized using xylene, followed by dehydration with an ethanol gradient, and rinsing with PBS twice. Molded samples were sectioned at 4 microns thickness and were stained for 3 to 5 min with hematoxylin after which they were washed with acid alcohol (1%). Afterwards, tissue slices were dehydrated in ascending grades of ethanol and were stained for 5 min with eosin. The stained tissues were dehydrated again as mentioned above, treated with dimethylbenzene, and sealed with neutral gum and at the end; slides were pictured at the focal length of 200 μm, 100 μm, and 20 μm under a light-sheet microscope. The obtained data was analyzed by persons blind to treatments.

### Statistical analysis

Data are presented as mean ± standard deviation. One-way analysis of variance (ANOVA) was used to analyze the differences between the groups. This analysis was followed by Tukey’s Multiple Comparison Test. Mann-Whitney U was looked upon for histologic changes testing. Statistical analysis of data was carried out using SPSS statistic software (Version 17.0). A level of p < 0.05 was considered significant.

## Results and discussion

### Characterization of CaF_2_ nanoparticles

**Fig. 1:**
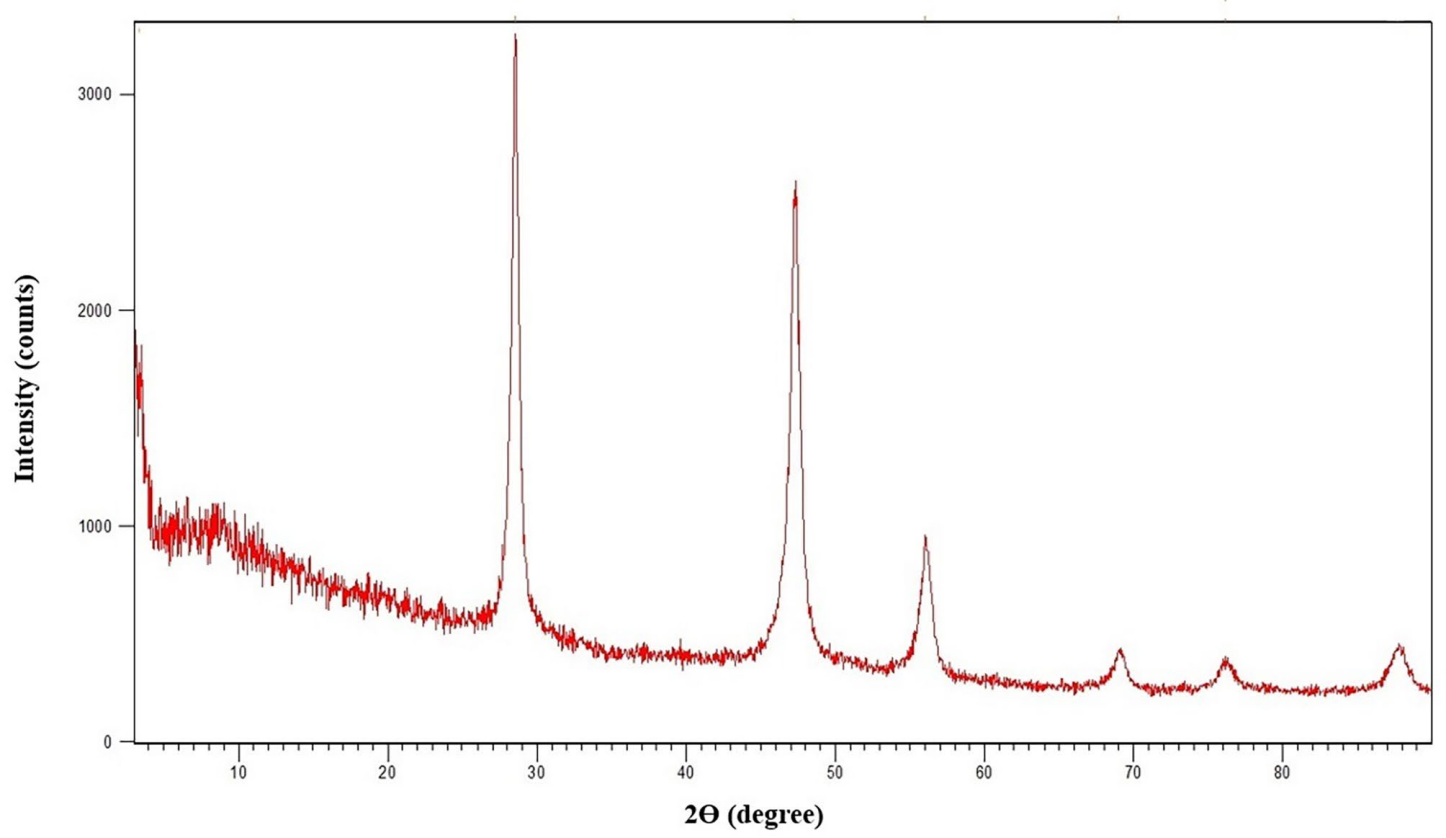
X-ray diffraction (XRD) spectrum of CaF_2_ nanoparticles

**Fig. 2:**
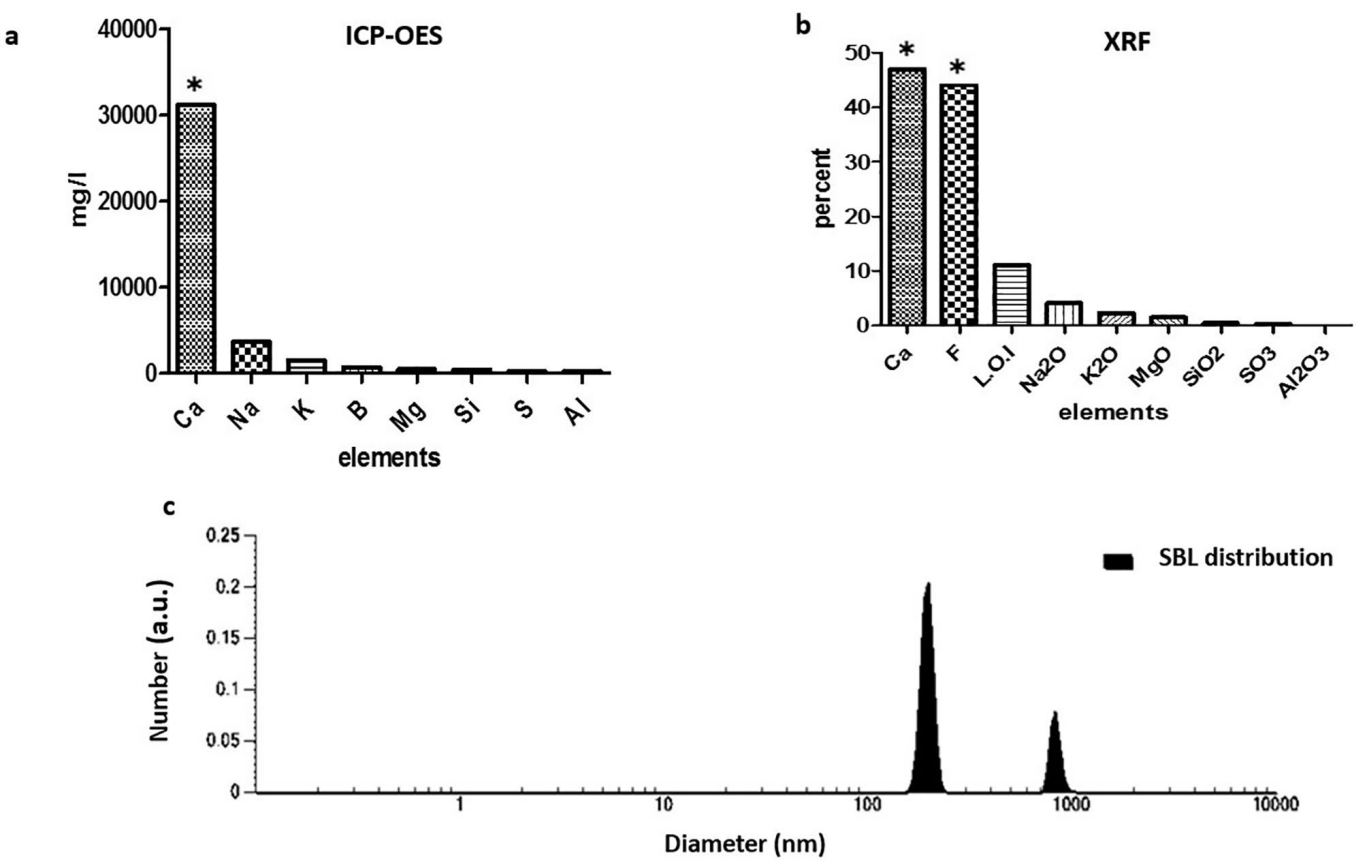
**a**) ICP-OES analysis. The amount of elements in CaF_2_ nanoparticles (ppm) **b**) XRF analysis. Percentage of elements in synthesized nanoparticle **c**) Hydrodynamic diameter of CaF_2_ nanoparticles measured by dynamic light scattering (DLS)

**Fig. 3:**
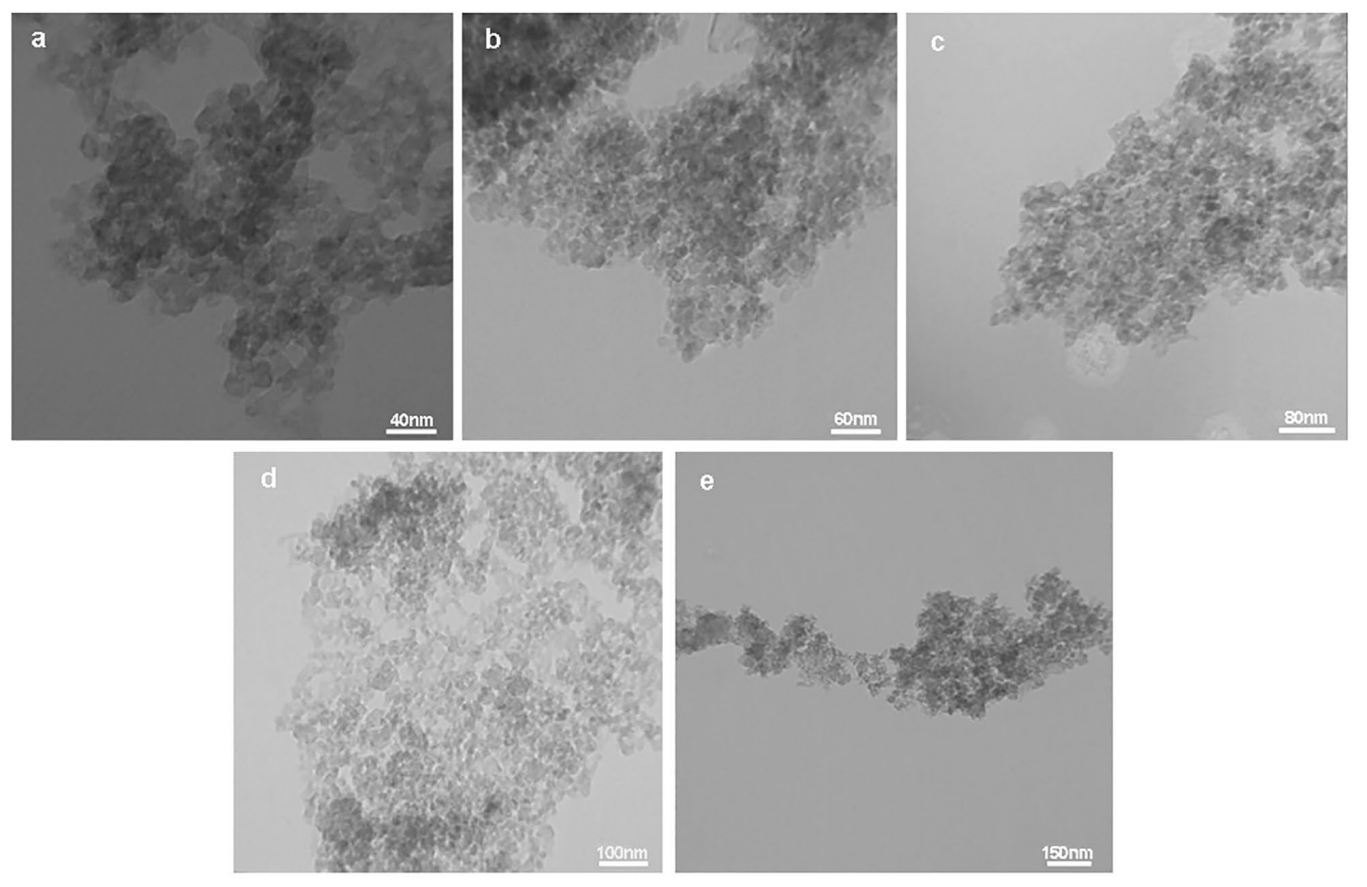
Transmission electron microscopy (TEM) images of CaF_2_ nanoparticles at different scale bars: **a**) 40 nm **b**) 60 nm **c**) 80 nm **d**) 100 nm **e**) 150 nm

The phase purity and crystallinity of the synthesized CaF_2_ NPs were identified by the X-ray diffraction technique. Figure 1 illustrates the XRD patterns of CaF_2_ nanoparticles. All of the diffraction peaks can be indexed to a face-centered cubic CaF_2_ phase, (space group 225:Fm3 m), which is in line with the standard values for cubic CaF_2_ (JCPDS Card no. 87–0971) [32], confirming our product is CaF_2_ nanoparticles in a complete crystalline structure. The XRD pattern shows broad peaks manifesting the small crystallite size of the produced sample. This result is according to those reported in previous studies [33]. According to Fig. 2a, the concentration of metals in nanoparticle solution was determined by ICP-OES. Results showed that there were a few analogous salts with calcium that were low in volumes such as sodium and potassium and the amount of the rest of the materials is almost zero, and this indicates the purity of the synthesized nanoparticle. As shown in Fig. 2b the XRF results indicated that the only constituents of synthesized nanoparticles are calcium and fluoride, and the amount of other elements is approximately zero. The results of the DLS assay showed that the synthesized sample size is between 100 and 200 nanometers, which is acceptable for the nanoparticle (Fig. 2c). The TEM images also confirmed the results of the DLS test and the nanoscale particles with spherical morphology were identified (Fig. 3). These images show conglomerates consisting of particles of less than 40 nm in size. These agglomerations can be attributed to both the intrinsic tendency of nanoparticles to the formation of agglomerates and the quality of sample preparation for TEM assay.

### Growth and body weight and tibial and femoral bone length

**Fig. 4:**
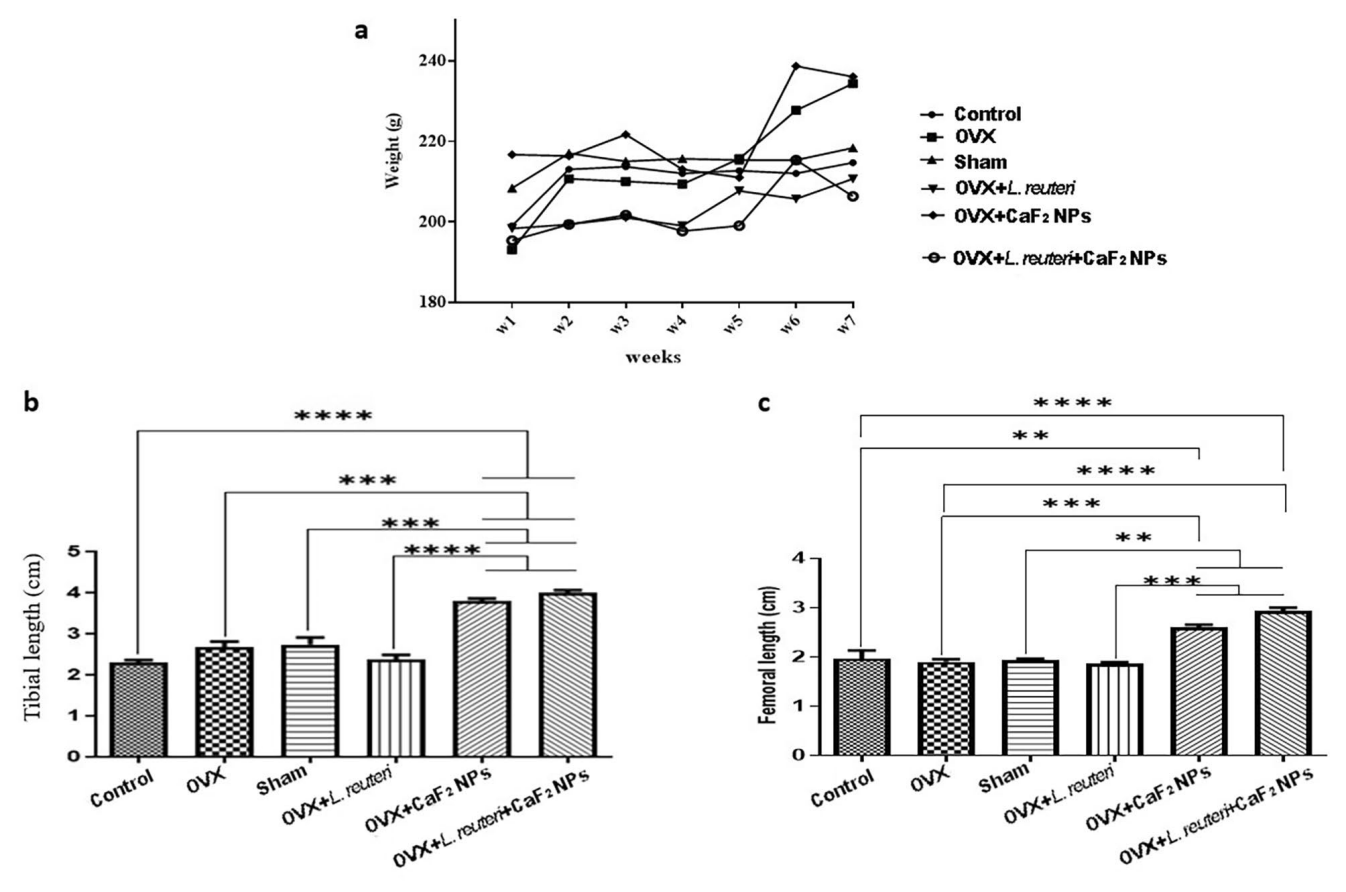
**a**) Average weight of all groups during different weeks of study **b**) tibial bone length **c**) femoral bone length. (** P < 0.01, *** P < 0.001, **** P < 0.0001 based on oneway ANOVA, followed by Tukey’s Multiple Comparison Test)

As shown in Fig. 4a, the weight diagram’s slope first went upward, then turned slightly downward, and then finally, their weight stabilized and maintained a slight ascending slope until the end of the trial. The reason why there is weight fluctuation at the beginning of the experiment can be the unfamiliarity of rats with the test environment and the stress caused by gavage, therefore weight fluctuation at the beginning of the study is quite normal. Bone length is a macroscopic factor that can determine decrement in bone atrophy after treatment [31]. As shown in Fig. 4 (b, c), in the control, OVX, sham, and OVX + *L. reuteri* groups, the length of the tibia and femur did not increase but in the OVX + CaF_2_ NPs and OVX + *L. reuteri* + CaF_2_ NPs, the length of the tibia (P < 0.001) and femur (P < 0.01) significantly increased compared to other groups. Therefore, our results show that *L. reuteri* alone has no effect on increasing bone length. However, previous studies indicated that oral administration of *L. reuteri* in male mice enhances bone density [34]. Another study also showed the beneficial effect of *L*. *reuteri* on increasing bone density in female mice, under an inflammatory status [35]. Fluoride ion has the therapeutic ability for osteoporosis healing and promoting osteoblast activity [36]. In this study for the first time, we investigated the influence of CaF_2_ nanoparticles regiments on osteoporosis. According to our results, the difference in bone growth between the two groups receiving CaF_2_ nanoparticles (group 5 and group 6) and other groups is statistically significant and this demonstrates that the synthesized nanoparticles can control induced osteoporosis in rats and increase bone length. A study also manifested that deficiency of calcium and fluoride can lead to adverse effects on the bone and induce bone loss [37].

### Histological slides of rats’ tissues

In the present study, we investigated the histological effects of *L. reuteri* and CaF_2_ NPs supplementation on kidney, liver, and bone tissues of rats using the H&E staining method.

#### **CaF**_**2**_**NPs and L. reuteri treatment represent an increase in bone tissue regenerative cells**

**Fig. 5:**
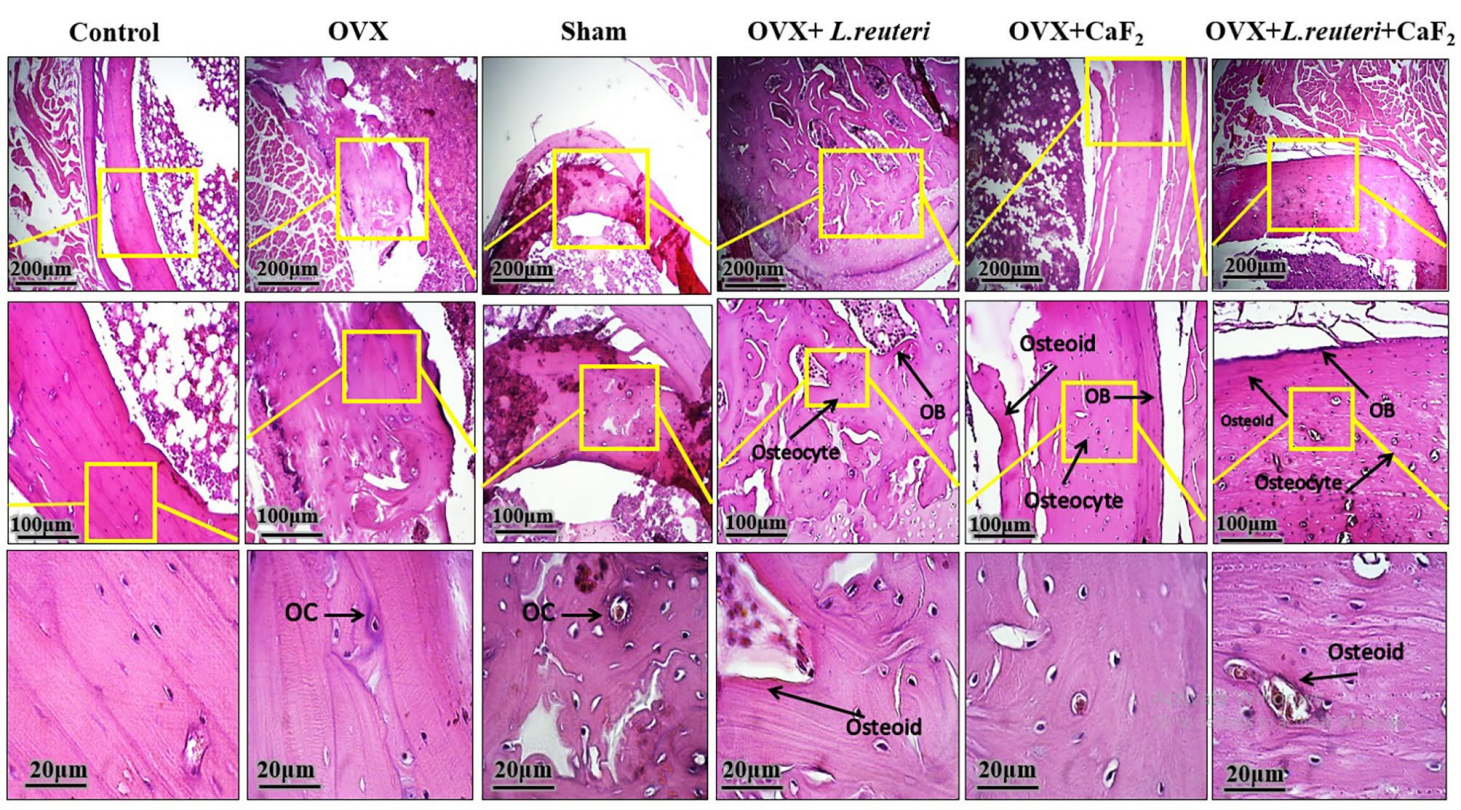
The histological light microscopy images of H&E stained sections in tibia bone of rats

Bone histopathology slides (Fig. 5), show the cross-section of rat tibia bone stained with H&E. In the images of bone longitudinal sections of OVX + *L. reuteri* and OVX + CaF_2_ NPs groups, it is clear that the population of osteoblasts and osteocytes and newly generated osteoid (unmineralized bone matrix) has significantly increased compared to the OVX and sham groups, and this increase in a cell population can affect the density and longitudinal growth of bone tissue. Also, by evaluating the bone images of the OVX and sham groups, it was found that part of the bone tissue is lighter in terms of coloration, which is due to the decrease in dense areas of bone, and this is the result of the decrease in calcium deposition in the tissue and reduction of activity of osteoblasts and the rate of conversion of osteoblasts to osteocytes. By investigating the bone tissue images of the control and Ovx + probiotic + CaF_2_ NPs groups, bone tissue cells including osteocytes and osteoblasts were observed in dense bone tissue. In addition, the images showed that the thickness of bone tissue in the treated group was largely similar to the control group in terms of the number of bone regenerating cells. This fact largely confirms the effectiveness of the treatments. Also, a study in which OVX mice were treated with *L. reuteri* for 4 weeks, showed that femur and vertebral trabecular bone volumes are similar to ovary intact control mice and *L. reuteri* treatment suppresses bone resorption and loss associated with estrogen deficiency [18]. Another study on women between the ages of 75–80 who received an oral dose of *L. reuteri* showed a slight reduction in bone loss [15]. Sufficient intake of Ca is important for bone health and reduced dietary intake of Ca is related to decreased bone mass and leads to osteoporosis. A study showed that feeding of Ca and vitamin D dietary supplements has an anti-osteoporotic effect in OVX rats and leads to inducing bone formation and abolition of bone loss [38]. The positive effects of the probiotics are associated with the high content of dietary Ca and the high levels of supplemented probiotics. Probiotics indicated beneficial effects on bone metabolism via various mechanisms with extraordinary results in the animal model. The studies also demonstrated that consuming probiotics by postmenopausal women with low bone mass density increases mineral bioavailability including Ca, resulting in increasing bone mass density. Some mechanisms cause an increase in the bioavailability of the minerals, including (1) hydrolyzing glycoside bond food by probiotics in the intestines; (2) enhancement of solubility of minerals because of production of short-chain fatty acids; (3) decreasing intestinal inflammation followed by increasing bone mass density; (4) producing phytase enzyme by bacteria to overcome the effect of mineral depressed by phytate [39]. Therefore, treatment with a combination of probiotics and calcium nanoparticles is likely to have a greater effect on increasing bone density and reducing the degree of bone loss, as observed in the present study.

#### No detrimental effects were found in the liver tissue from CaF_**2**_**NPs treatment**

**Fig. 6:**
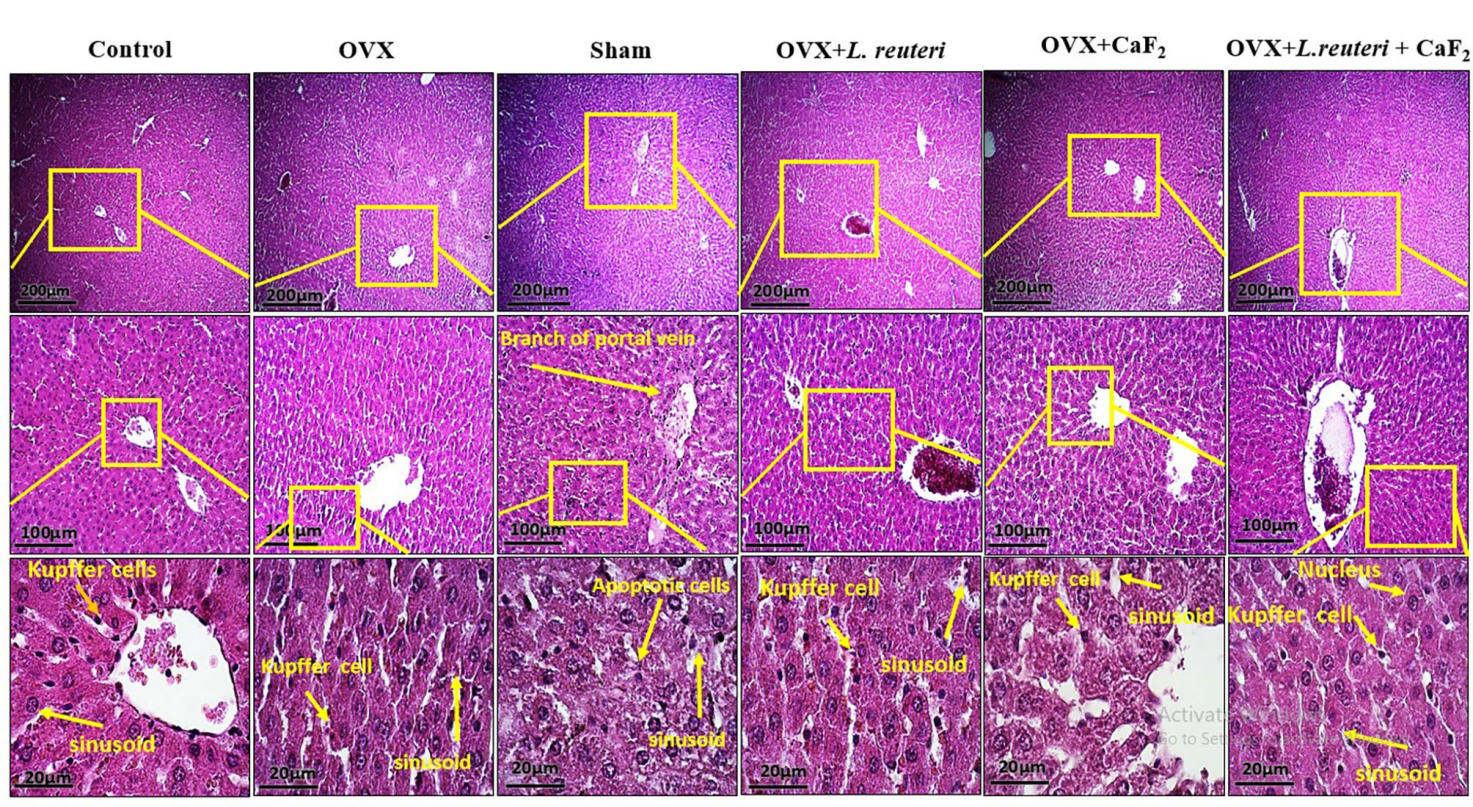
The histological light microscopy images of H&E stained sections in liver of rats

According to histological images of OVX + *L. reuteri* and OVX + CaF_2_ NPs groups, it was found that hepatic cells had a normal appearance (Fig. 6). The percentage of cells with apoptosis in these two groups was about 5%. These results indicate that gavage of *L. reuteri* and CaF_2_ NPs did not have a detrimental effect on liver tissue function. In OVX and sham groups, it was also observed that the percentage of apoptotic hepatocytes was not different from the control group and was about 5%. These results suggest that postmenopausal osteoporosis modeling has no destructive effect on liver tissue. In the OVX + *L. reuteri* + CaF_2_ NPs group, hepatocytes were observed in the form of normal and hepatic plates. Hepatic sinusoids from the lobule to the central vein of the lobule were observed at intervals between the hepatic plates. A hepatic triad was observed around the lobule and in the center of each central venous lobule. The percentage of apoptotic hepatocytes in this group was less than 5%. No nanoparticle deposition was observed in liver tissues.

#### The glomeruli and medulla of the treatment receiving groups were intact and normal

**Fig. 7:**
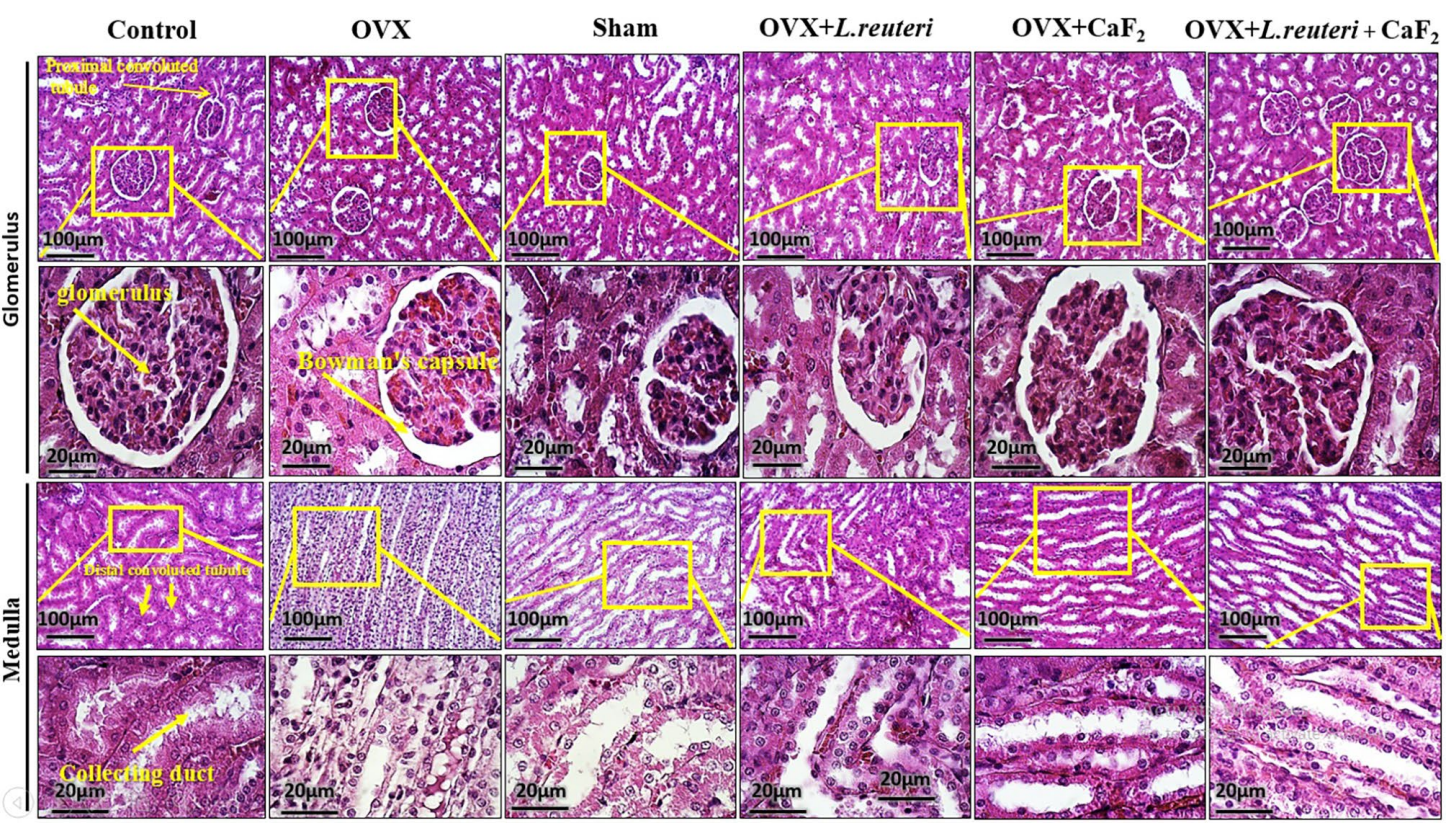
The histological light microscopy images of H&E stained sections in kidney of rats

As shown in Fig. 7, histological renal slides show the longitudinal section of rat kidney tissue. In the three main experimental groups, including OVX + *L. reuteri*, OVX + CaF_2_ NPs, and OVX + *L. reuteri* + CaF_2_ NPs groups, there were no glomerular disease states and the capsule spaces were normal. The percentage of lymphocytes in the tissues in treated groups did not change compared to the control group. In proximal tubules and distal tubules, epithelial cells were observed normally. These results indicate that the gavage of *L. reuteri* solution and synthesized nanoparticles did not have a detrimental effect on renal tissue function. Based on tissue slides of OVX and sham groups, it was found that the size of renal glomeruli did not change compared to normal and a slight hemorrhage was observed at some points inside Bowman’s capsule. In these two groups, there was no change in the number of lymphocyte cells in the tissue compared to the control group. Also in proximal tubules and distal tubules, epithelial cells were observed normally. These results indicate that the infertility modeling and ovariectomy did not have a negative effect on kidney tissue. Moreover, no nanoparticle deposition was observed in the kidney tissues of the treatment groups.

### Serological results

After 6 weeks of treatment, blood was collected from all groups of rats and after clotting at room temperature for 5 min serum samples were centrifuged for 10 min at 4000 rpm at 4 ˚C [18] and the supernatant above blood cells was collected for the detection of biochemical markers, including estrogen, calcium, vitamin D, and ALP.

#### CaF_2_ NPs and L. reuteri each alone raised the level of serum estrogen level to some extent, but the simultaneous treatment was able to create a significant difference

**Fig. 8:**
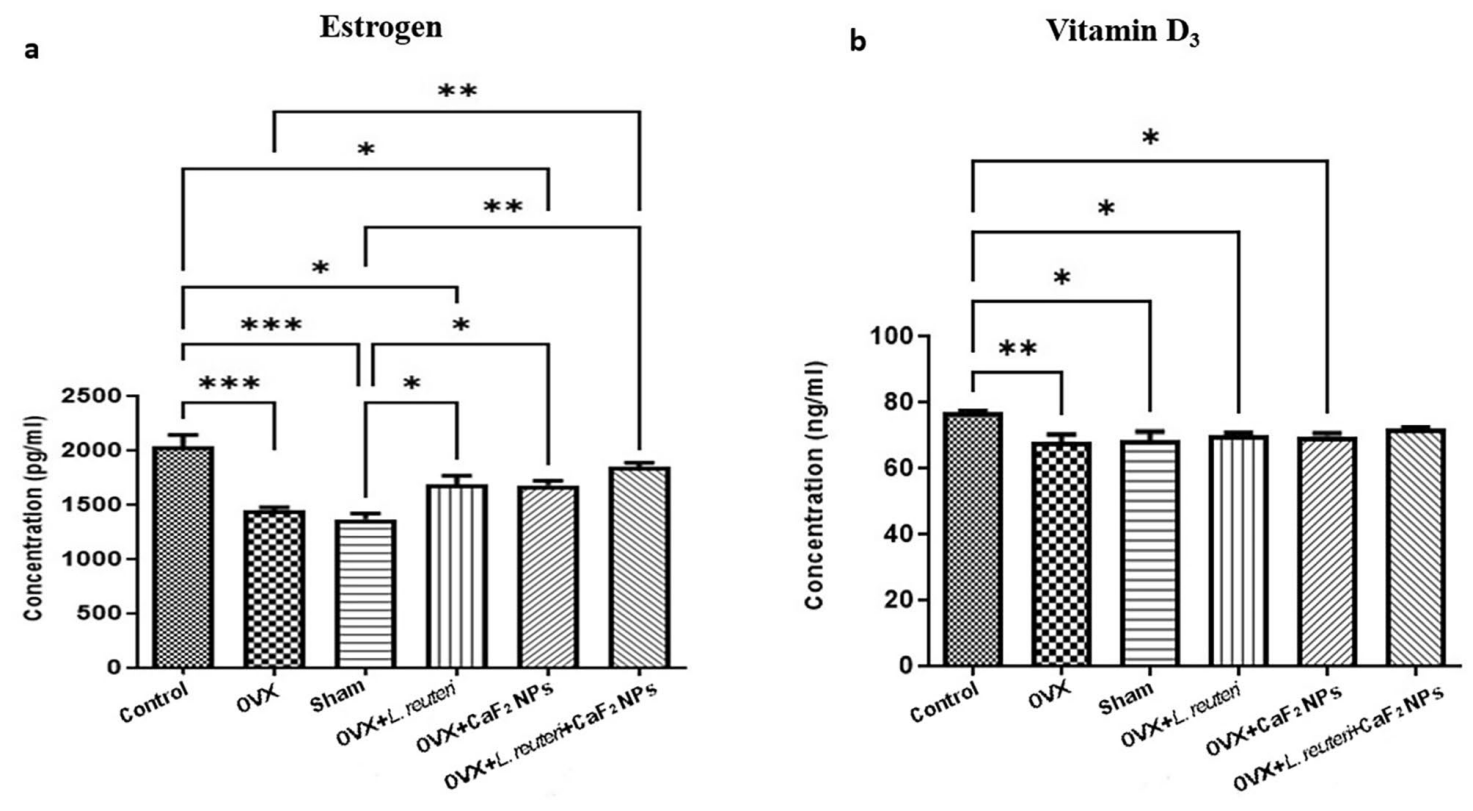
Evaluation of biochemical parameters in experimental groups. **a**) serum estrogen level. **b**) serum vitamin D3 level. (* P < 0.05, ** P < 0.01, *** P < 0.001 based on oneway ANOVA, followed by Tukey’s Multiple Comparison Test)

Estrogen influences bone tissue through the different mechanisms, including (i) inducing the generation of calcitonin, so preventing bone resorption, (ii) reducing the sensitivity of bone mass to parathyroid hormone, so decreasing bone resorption, (iii) diminishing the calcium excretion from the kidney, (iv) increasing calcium resorption via the intestine, and (v) direct effects on bone through the estrogen receptors [40]. Estrogen deficiency disrupts the normal bone turnover cycle at menopause and causes an irregular increase in bone resorption as compared with deposition [40]. In this study we assayed alters in estrogen levels in OVX rats under treatment with *L. reuteri* and CaF_2_ NPs. According to our results, as shown in Fig. 8a, in the OVX group in which rats underwent ovariectomy and didn’t receive any treatment, the amount of estrogen has significantly decreased compared with the control group (P < 0.001), and this is also true for the sham group which received a blank solution (P < 0.001). In OVX + *L. reuteri* and OVX + CaF_2_ NPs groups, estrogen levels increased almost the same amount, which was significant compared to the groups without treatment (P < 0.05). The combination therapy in the OVX + *L. reuteri* + CaF_2_ NPs group also showed a significant increase in estrogen levels compared to the sham and OVX groups (P < 0.01). The predominant metabolism of estrogens, including hydroxylation and conjugation, takes place in the liver. Conjugated estrogens go through the enterohepatic circulation to the bile and ultimately pass into the small intestine, where they are deconjugated by the gut microbiome and are partially reabsorbed, and enter the circulation through the portal vein [41]. According to a hypothesis, this enterohepatic recirculation impacts the systemic levels and half-life of estrogens in men and postmenopausal women, and probably during the luteal phase in premenopausal women. The diversity of the gut microbiome could influence systemic estrogen levels by different processes [42]. *L. reuteri* is one of the indigenous bacteria in the gastrointestinal tract of humans and animals [43]. Previous studies have indicated the modulatory impacts of *L. reuteri* on the microbiotas of piglets, rodents, and humans [17]. Therefore, according to these findings, we concluded that *L. reuteri* probiotic by modulating the gut microbiome may regulate serum estrogen levels. In addition, a more recent study in agreement with our results showed that calcium supplementation elevated the estradiol content level in OVX rats [44]. They proposed that the beneficial impacts of calcium supplementation on bone loss may be partially associated with the enhancement in the level of estrogen and stimulation of the changes in levels of metabolite, resulting in increasing the bone mineral density.

#### Simultaneous CaF_2_ NPs and *L. reuteri* treatment had a slight impact on the serum vitamin D_3_ levels regulation

The deficiency of serum vitamin D levels among postmenopausal women with osteoporosis is widely prevalent [45]. As shown in Fig. 8b, our results also showed a significant reduction in serum vitamin D_3_ levels in the OVX (P < 0.01), sham (P < 0.05), OVX + *L. reuteri* (P < 0.05), and OVX + CaF_2_ NPs (P < 0.05) groups compared with the control group. Treatment with *L. reuteri* and CaF_2_ NPs alone in our study didn’t show a significant benefit for increasing serum vitamin D_3_ levels in OVX rats. However, the combination therapy in the OVX + *L. reuteri* + CaF_2_ NPs group showed a slight increase in serum vitamin D_3_ level, which, perchance if the test time was longer, the level of vitamin D_3_ would even reach almost normal levels. In line with our results, previous findings showed that circulating 25-hydroxyvitamin D level increases in response to oral *L. reuteri* NCIMB 30,242 probiotic supplementation [46]. Another study also suggested that prebiotic supplementation can increase provitamin D_3_ biosynthesis [47]. Moreover, according to previous studies receiving calcium can increase the mean serum concentration of 25-hydroxyvitamin D_3_ [48]. Jorde et al. also reported that in the females, which have low vitamin D intake (< 7 µg/d) there is a significant increase in serum 25-hydroxyvitamin D_3_ level with increasing calcium intake [49].

#### Serum calcium levels in the receiving L. reuteri and combination of L. reuteri and CaF_2_ NPs treatment groups significantly decreased

**Fig. 9:**
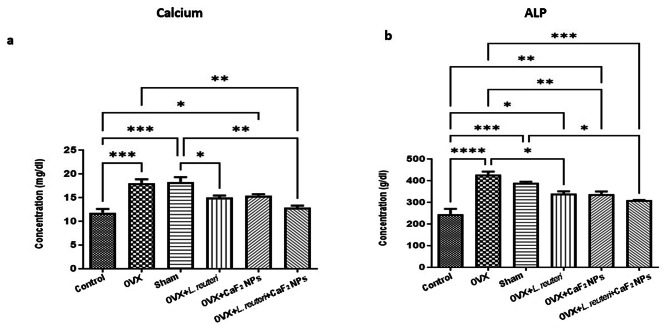
Evaluation of biochemical parameters in experimental groups. **a**) serum calcium level. **b**) serum ALP level. (* P < 0.05, ** P < 0.01, *** P < 0.001, **** P < 0.0001 based on oneway ANOVA, followed by Tukey’s Multiple Comparison Test)

According to our results (Fig. 9a), the OVX and sham groups, which are osteoporosis models, showed a significant increase in serum calcium levels compared with the control group (P < 0.001). Previous studies also showed that in the OVX mouse, intestinal calcium malabsorption occurs in postmenopausal osteoporosis, and ovariectomy caused a significant increase in fecal and urinary calcium and a significant decrease in calcium absorption [50]. Interestingly, enhanced calcium absorption by some probiotic bacteria such as *Lactobacillus salivarius* was indicated in Caco-2 cells [39]. *L. reuteri* probiotic can also increase calcium uptake by elevating calcium solubility [39]. Another study on *L. reuteri, Lactobacillus casei*, and *Lactobacillus gasseri* reported more calcium absorption and 35% higher bone weight between the probiotic-fed group compared with the control group in rat models [51]. Our results also showed *L. reuteri* in OVX + *L. reuteri* group significantly decreases serum calcium levels compared to the sham group (P < 0.05), which may be due to the increasing calcium absorption. CaF_2_ NPs alone in the OVX + CaF_2_ NPs group showed no significant effect on decreasing the serum calcium level compared to the sham and OVX as osteoporosis model groups. However, in the OVX + *L. reuteri* + CaF_2_ NPs group a significant reduction in the serum calcium level was observed compared to the OVX and sham groups (P < 0.01). By comparing the combination treatment group and the control group and considering their similarity in serum calcium levels, it was concluded that the combination therapy had a significant effect on calcium absorption and the return of serum calcium levels almost to the normal level. Probably, the presence of fluoride ions in this group, in addition to probiotic, helps to increase calcium absorption. Stamp et al. suggested that fluoride therapy in osteoporosis reduces the total calcium and ionized calcium concentrations, which may be due to the ‘movement’ of calcium from the circulation into tissues, such as the bone [52]. Another study also indicated that fluoride caused the reduction of calcium concentration in the serum of female rats resulting in calcium transportation to the intercellular fluid and deposition in bones and teeth [53].

#### Serum alkaline phosphatase (ALP) levels significantly decreased in the combination L. reuteri and CaF_2_ NPs treatment group

ALP is a membrane-bound glycoprotein that presents in many tissues in the body, with more concentrations in osteoblasts and in the liver. According to previous findings, ALP was identified as a marker of bone formation; although, more recent studies have proposed that ALP should be considered as a marker of bone turnover rather than bone formation [54–56]. Our results of serum ALP values in the OVX and sham groups showed a significant increase compared with the control group (P < 0.0001 and P < 0.001 respectively). A study in line with our results indicated that serum ALP level increases in the OVX-induced osteoporosis [57]. Skeletal system diseases associated with enhanced osteoblast activity and bone remodeling cause elevation of ALP level [58]. Previous studies showed that multispecies probiotic supplementation has beneficial effects on bone health due to reducing the rate of bone turnover. A recent randomized double-blind placebo-controlled clinical trial showed that ALP was significantly decreased in postmenopausal women after taking probiotic supplementation [59]. Our study also demonstrated that receiving *L. reuteri* probiotic significantly decreased the level of ALP compared with the OVX group (P < 0.05) (Fig. 9b). Another study also manifested that intervention with *L. reuteri* reduced ALP and led to diminished bone turnover [18], which is in agreement with our study. Also, group 5, which received CaF_2_ NPs, showed a significant decrease in serum ALP level compared with the OVX group (P < 0.01). Since appropriate doses of fluoride induce bone formation by osteoblastic stimulation, we concluded that the intake of CaF_2_ NPs in our study increases bone formation rate resulting in reducing serum ALP level in OVX-induced osteoporosis. Moreover, combination therapy with *L. reuteri* probiotic and CaF_2_ NPs was more effective and significantly reduced ALP levels compared with OVX and sham groups (P < 0.001 and P < 0.05 respectively).

This research was not in the absence of its shortcomings and limitations. Limitations of this work include the small sample size in each group (n = 3), the use of only one dose of the nanoparticle, and the short duration of the experiment. We suggest that long-term follow-up on a larger scale with different doses of CaF2 NPs alongside L. reuteri are needed to determine the effectiveness of these two substances simultaneously and individually.

## Conclusion

In this study, for the first time, we used combined dietary supplementation with *L. reuteri* and CaF_2_ nanoparticles for attenuation of bone loss in OVX-induced osteoporosis. According to our results, daily simultaneous supplementation of low doses of *L. reuteri* and CaF_2_ NPs for 6 weeks significantly increased the length of the tibia and femur. The histological images of rat tibia tissue also showed a significant increase in the population of osteoblasts and osteocytes and increased newly generated osteoid. Interestingly, no adverse side effects on the kidney and liver were observed in the treatment groups. In addition, the combination therapy also had a beneficial effect in reducing serum Calcium and ALP levels and increasing serum Estrogen and Vitamin D3 levels. Therefore, this combined supplementation could be a new and promising approach to reducing the symptoms of post-menopausal osteoporosis. Further long-term investigations on a larger scale are needed to dissect the mechanisms and to evaluate the usefulness of simultaneous therapy with *L. reuteri* and CaF_2_ NPs for preventing bone loss in osteoporosis.

## Data Availability

All data generated or analyzed during this study are included in this published article.
